# Low-coverage sequencing in a deep intercross of the Virginia body weight lines provides insight to the polygenic genetic architecture of growth: novel loci revealed by increased power and improved genome-coverage

**DOI:** 10.1016/j.psj.2022.102203

**Published:** 2022-10-01

**Authors:** T. Rönneburg, Y. Zan, C.F. Honaker, P.B. Siegel, Ö. Carlborg

**Affiliations:** ⁎ Department of Medical Biochemistry and Microbiology, Uppsala University, Uppsala, Sweden; † Department of Animal and Poultry Sciences, Virginia Polytechnic Institute and State University, Blacksburg VA, USA

**Keywords:** QTL mapping, low-coverage sequencing, body weight, advanced intercross line

## Abstract

Genetic dissection of highly polygenic traits is a challenge, in part due to the power necessary to confidently identify loci with minor effects. Experimental crosses are valuable resources for mapping such traits. Traditionally, genome-wide analyses of experimental crosses have targeted major loci using data from a single generation (often the *F_2_*) with individuals from later generations being generated for replication and fine-mapping. Here, we aim to confidently identify minor-effect loci contributing to the highly polygenic basis of the long-term, bi-directional selection responses for 56-d body weight in the Virginia body weight chicken lines. To achieve this, a strategy was developed to make use of data from all generations (*F_2_*–*F_18_*) of the advanced intercross line, developed by crossing the low and high selected lines after 40 generations of selection. A cost-efficient low-coverage sequencing based approach was used to obtain high-confidence genotypes in 1Mb bins across 99.3% of the chicken genome for >3,300 intercross individuals. In total, 12 genome-wide significant, and 30 additional suggestive QTL reaching a 10% FDR threshold, were mapped for 56-d body weight. Only 2 of these QTL reached genome-wide significance in earlier analyses of the *F_2_* generation. The minor-effect QTL mapped here were generally due to an overall increase in power by integrating data across generations, with contributions from increased genome-coverage and improved marker information content. The 12 significant QTL explain >37% of the difference between the parental lines, three times more than 2 previously reported significant QTL. The 42 significant and suggestive QTL together explain >80%. Making integrated use of all available samples from multiple generations in experimental crosses are economically feasible using the low-cost, sequencing-based genotyping strategies outlined here. Our empirical results illustrate the value of this strategy for mapping novel minor-effect loci contributing to complex traits to provide a more confident, comprehensive view of the individual loci that form the genetic basis of the highly polygenic, long-term selection responses for 56-d body weight in the Virginia body weight chicken lines.

## INTRODUCTION

Quantitative traits remain difficult to analyze and break down into their component loci (Flint and Mott, 2001). Effect sizes of individual loci often explain a very small fraction of the phenotypic variance (Boyle et al., 2017 and references within)–often much smaller than environmental effects–and are regularly dependent on the genetic background (Pettersson et al., 2011; Mackay, 2014; Forsberg et al., 2017; Zan and Carlborg, 2020). Experimental populations are valuable resources for studying quantitative traits, and by reducing confounding factors such as environmental noise, they have provided a clearer view on the genetic architecture of a wide range of complex traits (Andersson, 2001; Flint and Mott, 2001; Andersson and Georges, 2004). Examples include shank-length in mice (Castro et al., 2019 ), longevity in *Drosophila melanogaster* (Curtsinger and Khazaeli, 2002), and oil content in corn (Hopkins, 1899; Dudley, 2007).

Although quantitative trait loci (**QTL**) studies in experimental crosses have high power, resolution is limited due to the extensive linkage disequilibrium (**LD**) introduced by the crossing design. This meant that, historically, it was possible to use the sparse or very sparse marker maps available at the time without compromising the resolution much further, as resolution was primarily gated by the number of recombinations. Still, this resulted in regions of the genome with less coverage and, in some cases, missing data on small chromosomes and/or chromosomal ends (Mackay, 2001). Similarly, it was not uncommon to have regions lacking markers informative for line origin when using experimental crosses between outbred founders (Andersson, 2001). To increase resolution and facilitate fine-mapping of detected QTL, follow-up studies in additional crosses have been performed. Generally, these have excluded regions outside of previously observed QTL, therefore leaving much of the genome without further study. As a result, these studies may be underpowered or lack the resolution to make inferences on the genetic architecture of the studied traits beyond a few large-effect loci (Flint and Mott, 2001).

More complete dissection of highly polygenic complex traits requires large and powerful studies. In natural populations such as humans, hundreds of thousands of individuals have been used to study highly polygenic model traits such as height (Lango Allen et al., 2010; Yang et al., 2010; Wood et al., 2014). In experimental populations, smaller populations are required to detect even minor-effect loci due to the higher power achieved from, for example, segregation of alleles at intermediate frequencies and greater control over environmental influences. Genotyping and imputation approaches based on low-coverage whole genome sequencing (**WGS**; Altshuler et al., 2000; Andolfatto et al., 2011; Zhang et al., 2015; Pértille et al., 2016; Whalen et al., 2018; Zan et al., 2019), have provided opportunities to reanalyze existing individuals generated for different purposes to perform integrated analyses, thus enabling greater insights into the contribution of loci with minor effects on the genetic basis of complex traits in existing experimental populations. The increased genome-wide marker coverage provided by WGS-based genotyping technologies also provides an extended coverage of regions outside of the current consensus linkage maps in species such as the chicken, where the major focus has been on the large chromosomes, leaving the microchromosomes largely unexplored (Groenen et al., 2000, 2009; Elferink et al., 2010).

The Virginia body weight lines of chickens were developed by long-term, bi-directional selection for a single trait—body weight at 56 d of age—resulting in a nine-fold difference between the low (**LWS**) and high (**HWS**) weight lines after 40 generations of selection (Dunnington and Siegel, 1996; Márquez et al., 2010 ; Dunnington et al., 2013 ). Genome-wide comparisons showed that the footprint of selection between LWS and HWS covers hundreds of loci across the genome (Johansson et al., 2010; Lillie et al., 2018, 2019). Efforts to identify which of these loci contribute to the observed responses include a series of experiments utilizing an intercross developed from individuals of generation S_41_ (n_HWS_ = 29, n_LWS_ = 30). Efforts include genome-wide mapping (Jacobsson et al., 2005; Carlborg et al., 2006; Wahlberg et al., 2009), as well as replication and fine-mapping studies (Besnier et al., 2011; Pettersson et al., 2011, 2013; Sheng et al., 2015; Brandt et al., 2017; Zan et al., 2017) on different generations in this population. Although these studies agree that the long-term responses are primarily from selection on a highly polygenic genetic architecture where most loci have small effects, the statistical support for individual loci is low. This study aims to overcome this deficiency of statistical power to facilitate the mapping of contributing minor-effect loci with confidence by re-genotyping and performing an integrated analysis of >3,300 individuals from generations *F_2_* to *F_18_* of the intercross of the Virginia body weight lines. Aims were to identify and map new QTL, confirm earlier reported loci, and explain more of the selection responses with individually significant loci than previously reported.

## MATERIALS AND METHODS

### Materials

All chickens described in this study were White Plymouth Rock originating from the Virginia body weight lines and their intercross. The procedures involving animals used in this experiment were carried out in accordance with the Virginia Tech Animal Care and Use Committee protocols.

The custom code, software versions, and software parameters used in this manuscript can be found in a dedicated Github repository at github.com/CarlborgGenomics/AIL-scan

The sequencing data generated for this project can be found in the NCBI sequence read archive, BioProject PRJNA788343.

### Sequencing

#### Founder-line Sequencing and Variant Calling

All 59 HWS and LWS founders of the AIL (n_HWS_ = 29 and n_LWS_ = 30) were whole-genome re-sequenced to ∼30X coverage (Guo et al., 2019). The obtained reads were mapped to the newest reference genome (GalGal6a, Genome Reference Consortium, 2018) using *BWA* (version 0.7.17; Li, 2013). Variants were called and filtered using GATK (McKenna et al., 2010) according to best-practices recommendations (DePristo et al., 2011; Auwera et al., 2013) modified to accommodate for nonmodel organisms.

#### Low-coverage Sequencing of Advanced Intercross Line Individuals

In addition to the 837 *F_2_* individuals sequenced by Zan et al. (2019), all remaining chickens from generations *F_2_*-*F_18_* of the AIL (total, n_F2-F18_ = 3,327, Table S2) were sequenced to ∼0.4X coverage using the strategy outlined by Zan et al. (2019). The obtained reads were mapped to the GalGal6 reference genome using *BWA* (version 0.7.17; Li, 2013). Variants were next called using a pipeline implemented using *bcftools* (version 1.9; Li, 2011), *samtools* (version 1.9; Li et al., 2009), *biopython* (version 1.70; Cock et al., 2009), and *cyvcf2* (version 0.10.0; Pedersen and Quinlan, 2017). Obtained variants were merged for each generation and filtered to only include polymorphisms present in the filtered set of founder genotypes described in the section above. Next, for each individual, only variants informative about the founder line origin (HWS/LWS) were kept and formatted for use as input to the *Stripes* genotyping pipeline (Zan et al., 2019).

### Genotype Estimation in the Virginia Body Weight Line AIL Using Deep-Stripes, a Pipeline for Founder-Line Genotype Estimation in Deep Intercross Populations

#### Deep-Stripes

*Deep-Stripes* was used to call founder line origin genotypes in 1 Mb bins across the genome of the *F_2_* to *F_18_* generations of the advanced intercross line (**AIL**). For this, high-coverage sequence data from the outbred founders of the population and low-coverage sequence data from the intercross individuals, generated as described below, were used.

*Deep-Stripes* is an extension of *Stripes* (Zan et al., 2019), a pipeline for founder-line genotype estimation using low-coverage sequencing data, adapted from *TIGER* (Rowan et al., 2015) for use in outbred intercross populations. In deep intercross populations from outbred founders, such as the AIL studied here, there is a generally lower and more variable density of founder-line informative markers. Here, we have further extended the *Stripes* pipeline to deep intercross populations, including updates to enhance stability and improve genotype calling quality in later generations.

*Deep-Stripes* updates are implementations of (a) reverting to hardcoded genotype emission thresholds in cases where the original nonlinear minimization procedure for determining these (Rowan et al., 2015) failed due to uniform ancestry across an entire chromosome, (b) a modified nonlinear minimization procedure improving convergence as well as defaulting to hardcoded parameters after 20 unsuccessful tries to determine the genotype emission thresholds, (c) a modified logic for comparing highly similar beta distributions to make results stable across computing platforms, and (d) automation of multiple rounds of genotype estimation for each individual.

#### Genotype Quality Control and Filtering

In order to detect incoherently called genotypes in low-information areas due to a delay of inferred crossovers to the end of such regions, *Deep-Stripes* implemented genotype estimation in both directions on the chromosome. Genotype estimation in this way with 2 window-sizes (50 and 200 markers) reduces the number of false positive crossovers in marker-dense areas resulting from the increased flat, per-window genotype estimation error rate in high marker density regions. The final genotypes were assigned by transforming the output from each of the four runs (2 directions × 2 window-sizes) described above to a genotype matrix. These contained estimated founder line genotypes for each individual in even-sized (1 Mb) bins across the genome. The process for this used the 4 matrices as follows: In bins where no recombination event was inferred, the genotypes were coded as numerical values (1, 0, −1) corresponding to the homozygote for founder-line 1, heterozygote and homozygote for founder-line 2, respectively. Recombination breakpoints were estimated with bp resolution. However, if one or more recombination events were detected, resulting in multiple genotypes being called in a 1 Mb window, the genotype was scored on a continuous scale from 1 to −1 by averaging the founder genotypes scores across the base pairs in the segment. Second, the genotype matrices from the forward and reverse runs were filtered by considering bins where estimates differed by more than one recombination event as uninformative and setting them to missing. This procedure was performed separately for the two window-sizes (50 and 200 markers). Third, the forward- and reverse-filtered genotype matrices obtained using 50 and 200 marker window-sizes were combined. This was done by using the genotype derived using 50 marker sliding windows if the marker density was below 20 markers/Mb and 200 if above. Bins with ambiguous genotypes were set to missing. These were defined as bins with genotype scores in the ranges [0.8, 0.2] and [−0.8, −0.2]. Finally, all bins genotyped in less than 100 individuals were set to missing.

### Analysis

#### QTL Mapping

QTL mapping was performed for body weight at 8 wk of age. To correct for generational effects, 8-wk body weights were standardized within each generation. This was done by subtracting the generation mean from each observation and then dividing it by the within-generation standard deviation. The genome scan was performed using the ‘scanone’ function from the package ‘qtl’ (Broman et al., 2003) in R (R Core Team, 2013) using Haley-Knott regression (Knott and Haley, 1992) with sex as a covariate. Significance threshold was obtained using permutations (n = 10,000), resulting in a 5% genome-wide significance of a Logarithm of Odds (**LOD**) score of 4.01.

#### Identification of Suggestive QTL using a FDR Approach

Suggestive QTL were here defined as those that reached a 10% false-discovery rate (**FDR**) threshold. First, LOD scores for markers across the genome were transformed into *P*-values (Peirce et al., 2006). They were then evaluated against a significance threshold adjusted for multiple testing using the Benjamini Hochberg procedure with a false discovery rate of 10%, as implemented in *statsmodels* (Benjamini and Hochberg, 1995; Seabold and Perktold, 2010).

#### Estimation of the Genetic Effect and Residual Variance Explained by the Mapped QTL

To estimate the residual variance explained by the QTL, we corrected for the sex effect using a linear model and fit either 1) all 12 significant or 2) all 42 suggestive and significant QTL using the *fitqtl* function in *r/qtl* on the residuals. This was done on the same individuals used in the QTL scan. Estimates for the residual variance explained by each QTL were obtained by fitting all significant and suggestive QTL jointly and using the SSv3 drop-one-term anova in the *fitqtl* function. Estimates for the effect on body weight in grams for each QTL were determined by fitting each QTL individually with sex as a covariate (Table S3). Due to the standardization of the phenotypes, the estimates were multiplied with the population-standard deviation to obtain an estimate in grams. The sum of these estimates was then expressed as a fraction of the between-line difference. The same was also done by fitting all QTL jointly (Table S4).

(1) $$y=SEX+\;\sum_{i=n}^{1}QTL_{i}$$

#### Estimating the Effect of Increased Genotyping Information Content on Statistical Power in QTL Analyses

The information content (**IC**) was calculated across the genome in the set of *F_2_* individuals that were common to this study and the earlier one of the *F_2_* generation (Wahlberg et al., 2009). The measure used was defined as (Knott et al., 1998):

(2) IC=VAR(a)+2VAR(d)

This was calculated at individual marker locations, as well as every cM, across the genome using the *a* and *d* indicator regression variables from Wahlberg et al. (2009). For the dataset from this study, the a and d indicator variables were calculated from the genotype estimates in each 1Mb bin as (Knott and Haley, 1992):

(3) $$a=P\left(Homozyote_{HWS}\right)-P\left(Homozygote_{LWS}\right)$$

(4) d=P(Heterozygote)

The information content was compared at the physical locations (Mb; Genome Reference Consortium, 2018) of the genotyped markers in Wahlberg et al. (2009) and across all tested locations in the 2 studies (every cM/Mb; Wahlberg et al., 2009; this study).

## RESULTS AND DISCUSSION

### Increased Coverage in the Genome-Wide Scan via Genotyping by Low-Coverage Sequencing

**Figure 1:**
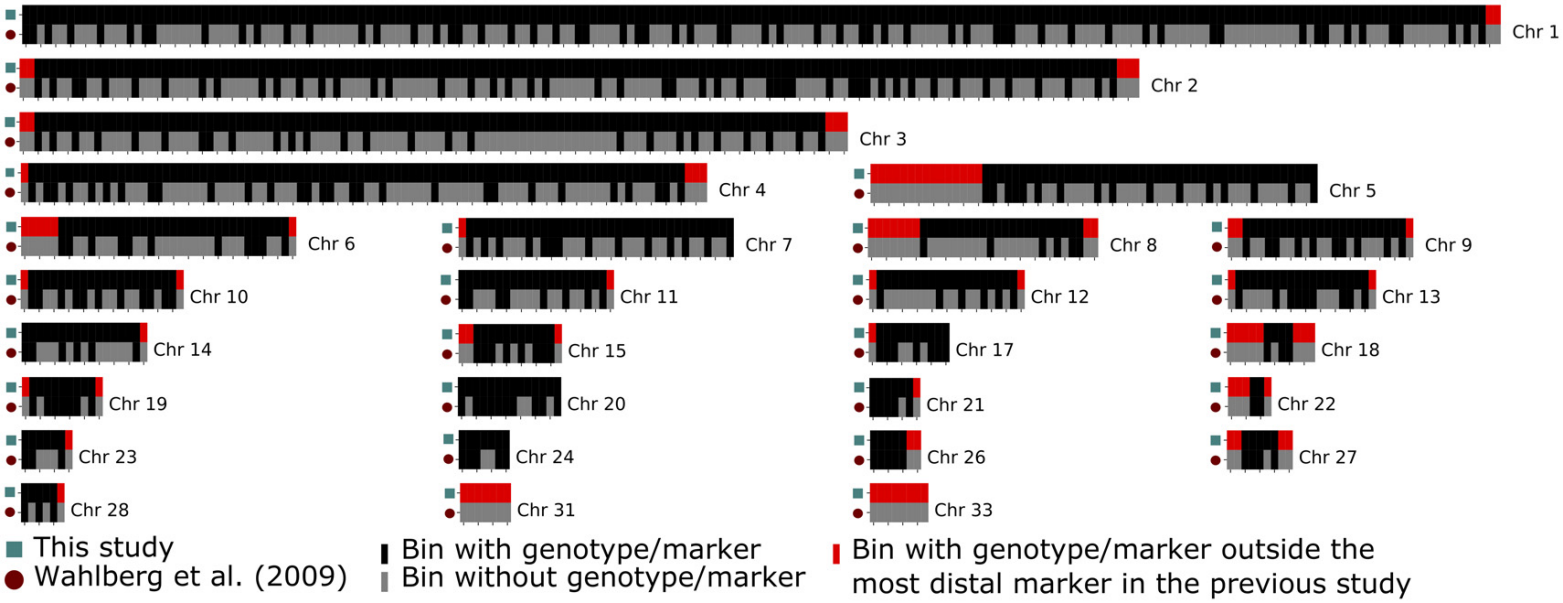
Genome coverage by the use of low-coverage sequencing data compared to an earlier *F_2_* genome scan (Wahlberg et al., 2009) based on 372 SNP and microsatellite markers. Black/grey colors indicate 1 Mb bins with/without genotypes and red highlights chromosome ends with new genotypes outside the outermost markers in Wahlberg et al. (2009).

After sequencing and variant calling, genotypes were estimated for n_F2-F18_ = 3,327 AIL individuals that passed quality control. The average density of informative markers across the genome was 102 markers/Mb. Because the founder lines are outbred and the individual AIL offspring descend from different founders, the number of informative SNPs varied among individuals and decreased over generations, as more ancestors contributed to the genotype of each individual. Founder-line genotypes were obtained for all of the 1,058 1Mb bins defined on the 33 largest chromosomes, identifying an average of 74 recombination breakpoints per Mb across all individuals before filtering. The accuracy of this imputation approach was previously validated on a subset of the individuals against experimentally determined SNP genotypes by Zan et al. (2019; see Figure 3) Compared to the previous genome-wide scan performed in the *F_2_* population by Wahlberg et al. (2009), an additional 100 Mb were covered (+27%) with markers, encompassing 2 small chromosomes (Chr31, Chr33), several previously uncovered scaffolds/unplaced segments and chromosome ends (Figure 1). Further, the information content at the tested locations across the genome also improved from an average of 0.77 (Wahlberg et al., 2009) to an average of 0.90 (Figure 3, panel F).

Since the founder lines were outbred, only a small fraction of the total markers genotyped for each individual are informative for line origin, averaging between 1.4 and 8% (4.7 ± 3.3%, on average >100.000 markers, Figure S1). While these are more than sufficient to impute founder-line haplotypes with confidence, the trade-off for this type of imputation is that it does not account for variation within the outbred lines, which limits detection power for QTL that do not differ between the lines or segregate in only one line. While this trade-off for confidence and power in detecting QTLs is a limitation, it is due to the imputation, and could be supplemented by alternative approaches on the same sequencing data for subsequent finemapping.

### More Individuals and Increased Genome-Coverage Facilitate Detection of New QTL

#### Comparisons to Earlier Mapped QTL from Genome Scanning of the AIL

**Figure 2:**
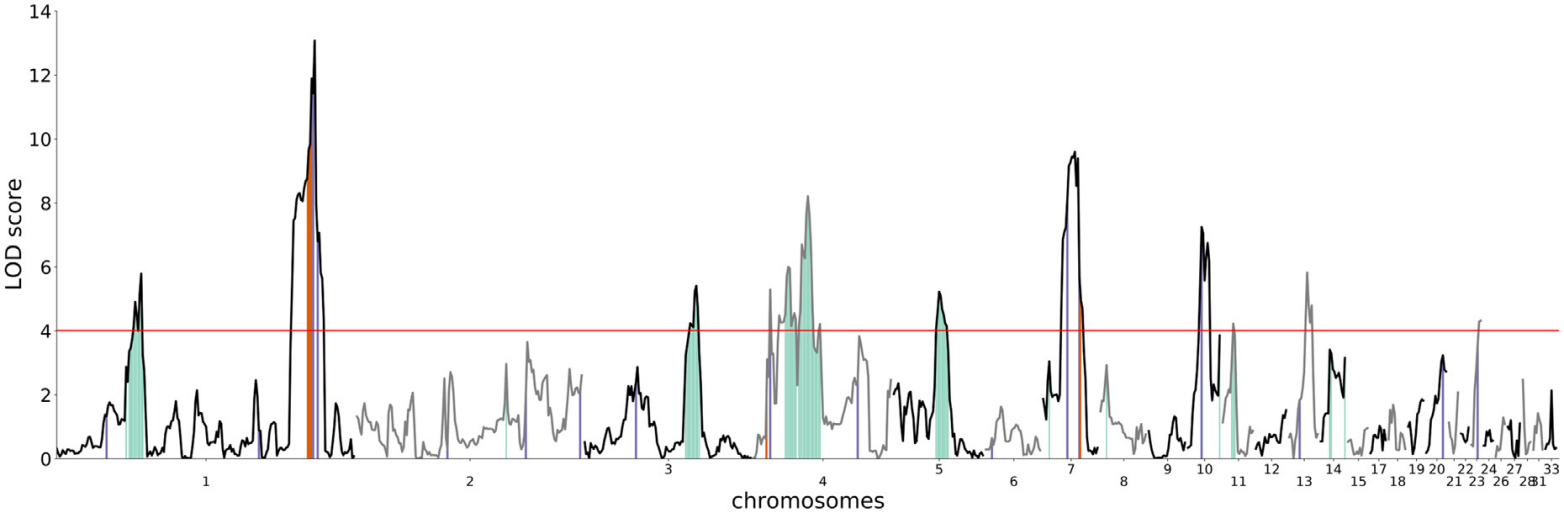
Genome-wide QTL scan for 56-d body weight in generations F to $F_{18}$ of the Virginia body weight lines AIL. The y-axis shows the statistical support for QTL as LOD scores and the x-axis the genomic location in Mb bins. The solid red horizontal line shows the genome-wide significance thresholds. Red vertical segments show the most recent earlier reported associations in genome-wide scans in $F_{2\;}$(Wahlberg et al., 2009), blue vertical segments indicate associations in the fine-mapping analyses in $F_{15}\;$(Zan et al., 2017). Green vertical segments indicate new suggestive QTL without a previous association within 15 Mb.

As illustrated in Figure 2, the 2+1 genome-wide significant and suggestive QTL in the most recent genome-scan of the $F_{2}$ generation of the Virginia line AIL (Table 1; Wahlberg et al., 2009) were also detected here. Further, nine additional QTL for this trait were also mapped with genome-wide significance (Figure 2; Table 1). At a 10% FDR-adjusted threshold for significance, a total of 42 QTL were identified (Figure 2; Table S3), that is, 30 QTL in addition to the 12 genome-wide significant QTL.

**Table 1:** Significant QTL for 56-day body weight and overlaps with earlier reported significant and suggestive QTL in this population.

Chromosome	Position (Mb)	a(SE)^1^	%var^2^	LOD^3^	QTL	Previous
1	56	19.6 (4.0)	0.43	5.79		
1	171	31.6 (4.1)	1.21	13.08	Growth1	Zan (2017), Wahlberg (2009)
3	74	20.3 (4.2)	0.31	5.41	Growth4	
4	11	20.7 (4.4)	0.28	5.29	Growth6	Zan (2017), Wahlberg (2009)
4	23	20.4 (4.0)	0.07	6	Growth7	
4	36	25.6 (4.2)	0.45	8.2	Growth7	
5	30	20.9 (4.3)	0.31	5.22	Growth8	
7	21	25.6 (4.0)	1.26	9.6	Growth9	Zan (2017), Wahlberg (2009)
10	9	22.3 (3.9)	0.27	7.25		Zan (2017)
11	7	13.2 (4.0)	0.32	4.22		
13	12	19.6 (3.9)	0.32	5.83	Growth10	
23	6	10.9 (4.3)	0.47	4.32		Zan (2017)
		a = 250.7				
		2a = 501.4				

1 Estimated effect size.

2 Percent of total variance explained.

3 Logarithm of Odds.

**Figure 3:**
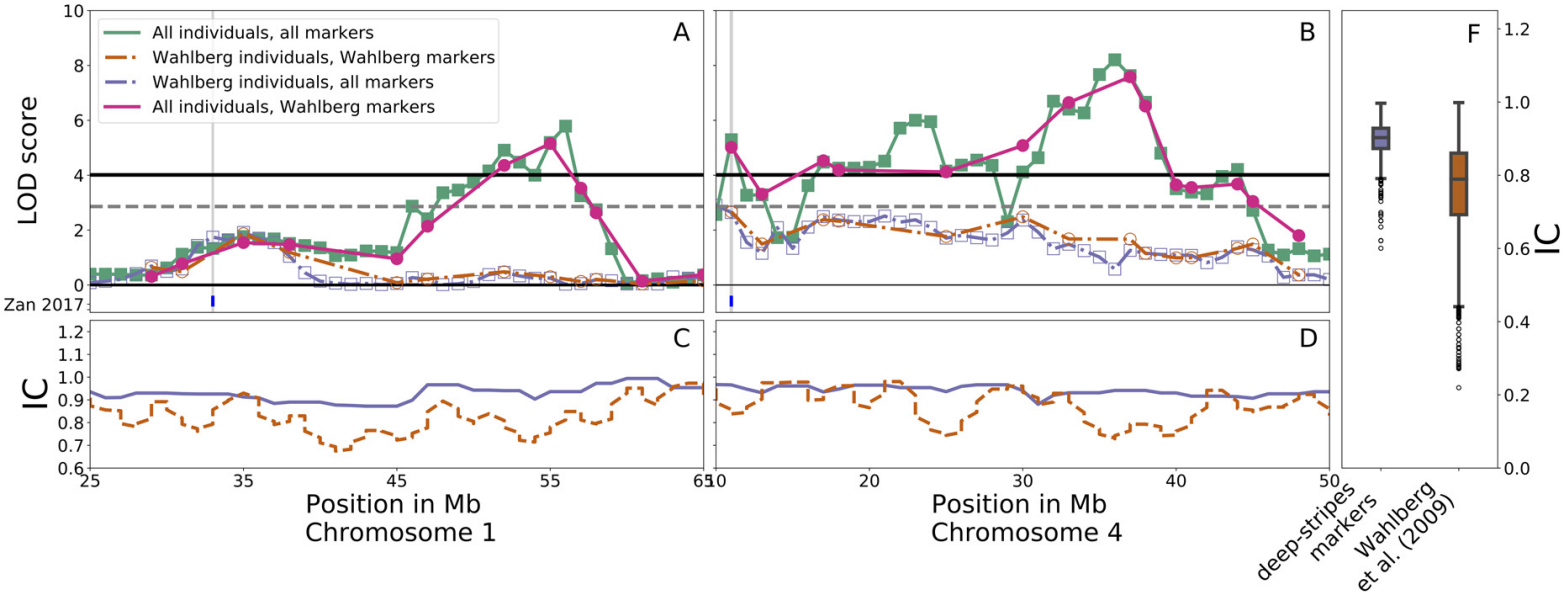
Comparisons to previous marker-sets from Wahlberg et al. (2009). Upper left and middle panels show the LOD score across selected peak regions on chromosome 1 (25–65 Mb) and chromosome 4 (10–50 Mb), using different selections of markers and individuals. Dashed/solid lines show the QTL scans using the *F_2_* individuals from Wahlberg et al. (2009). All individuals, solid round/empty square markers show QTL scans using markers from Wahlberg et al. (2009) / low coverage data. Bottom left and middle panels display information content across the same regions on Chromosome 1 and 4 for the markers used in Wahlberg et al. (2009) (orange, dashed) and the *deep-Stripes* markers (periwinkle, solid) across the individuals used by Wahlberg et al. (2009). The right panel summarizes information content for the same sets of markers and individuals, but across the 30 largest chromosomes.

### Overlap With Suggestive Regions From Fine-Mapping Analyses of the Virginia Line AIL

Several fine-mapping studies (Besnier et al., 2011; Sheng et al., 2015; Zan et al., 2017) have been performed in 1) 10 QTL-regions with significant and suggestive associations to 56-d body weights in the $F_{2}$ generation (Wahlberg et al., 2009) and 2) 99 selective sweep regions identified in comparisons of the HWS and LWS Virginia lines (Johansson et al., 2010). Here, 2 loci (located on Chr10, Mb 9, and Chr23, Mb 6) that were not part of the significant or suggestive QTL in the original $F_{2}$ genome-scan (Wahlberg et al., 2009) reached genome-wide significance when analyzed across the entire AIL (Figure 2, Table 1). Further, when comparing the 42 QTL significant at a 10% FDR threshold to 99 regions under selection evaluated by Sheng et al. (2015), 41 of these overlapped.

### More Individuals and Increased Marker Density Dissects a QTL on Chromosome 4 into Multiple, Independent Associated Regions

Previously, 2 regions on Chromosome 4 were implicated for body weight, with one of them, *Growth6*, reaching genome-wide significance for 56-d body weight in the latest genome scan of the *F_2_* generation (Wahlberg et al., 2009). The population used here provided sufficient power to replicate the previously reported QTL and confirm the association of another region, *Growth7*, that was previously reported as associated with body weight at different ages and growth traits (Figure 3, panel B, compares $F_{2}$ individuals from Wahlberg et al. (2009; dashed lines) to the AIL population (solid lines)).

In addition, the approach used here provided sufficient resolution to partition *Growth7* into three independent peaks (4/23, 4/36, 4/70 Chr/Mb). Figure 3, panel B, provides a comparison of the full AIL population (solid lines) with *deep-Stripes* inferred genotypes (empty squares, green) to the genotypes from the SNP and micro-satellite based marker panel used in Wahlberg et al. (full circles, pink) as an illustration of how the additional resolution facilitated separation of the 4/23 and 4/36 loci.

### Large Contributions by Mapped QTL to the Selection Response

The AIL was produced by intercrossing chickens from HWS and LWS after 40 generations of selection (S41), and the founders for the intercross differed more than 8-fold (1,341 g) in 56-d body weight (Table 1). Estimated from the AIL $F_{2}$-$F_{18}$, the 12 QTL reaching genome-wide significance explain 501.4 g (37.4%) of the difference between the parental lines (Table 1). This compares to the 159 g (12%) explained by the 2 significant QTL in Wahlberg et al*.* (2009). Together, the significant QTL mapped here/by Wahlberg et al. (2009) explained 8.3/5.2% of the residual phenotypic variance in the AIL *F*_8_ to *F*_18_ populations (Table 1). The 42 markers derived from the FDR-approach explain 14.6% of the residual phenotypic variance. This corresponds to 1,130 g (84.3%) of the difference between the founder lines using individually fitted estimates or 793 g (59%) of the difference between the founder lines using estimates from the joint fit.

### Concordance With Previous Estimates of Effect Sizes

The difference in effect size estimates for *Growth1* on Chromosome 1 between Wahlberg et al. (2009) and the approach used here were within the range of the standard error (34.2 ± 9.2 g and 31.6 ± 4.1 g, respectively). The effect size estimated here for *Growth6* on Chromosome 4 (20.7 ± 4.4 g) was lower than the previous estimate (36.3 ± 8.3 g). However, the current approach found 3 significant and 2 suggestive QTL on Chromosome 4. Of these, 2 significant and one suggestive QTL were within a QTL region (*Growth7*) reported in the first analysis of the *F_2_* generation (Jacobsson et al., 2005), but later showed no significant association with 56-d body weight in the extended analysis of the same individuals with a more comprehensive marker set by Wahlberg et al. (2009). In this study, these 3 + 2 QTL explained a total of 91.7 g suggesting a larger total contribution by Chromosome 4 than the earlier studies. In contrast, *Growth9* on Chromosome 7 had a reduced effect size estimate (25.6 ± 4.0 g), compared to the 43.2 ± 9 g estimated by Wahlberg et al*.* (2009).

### Additional Power in the Analyses Facilitates Detection of New QTL

#### A Novel QTL on Chromosome 1 Revealed by Combining Data Across Generations

A genome-wide significant QTL was mapped to 27 Mb on Chromosome 1 (Figure 3a; Table 1). This region was covered by multiple SNP and micro-satellite markers in the previous genome scans (Wahlberg et al., 2009; Figure 1). However, as these markers segregated in the founder lines, the estimation of founder-line QTL genotypes was less precise than the current approach utilizing low-coverage sequencing data (Figure 3c). To evaluate the potential contribution by either improved marker density or larger number of individuals to an increase in statistical power, the genome scan was performed on different subsets of the data. The first subset was $F_{2}$ individuals from Wahlberg et al. (2009), and second, the subset of bins containing SNP and microsatellite markers from the same study. Third, all combinations with the individuals and markers from this study were used. The results showed (Figure 3) that finding this QTL was driven by the integration of all generations in the AIL rather than the increase in marker density.

### New QTL Revealed in Regions With Poor Marker Coverage in the Genome

**Figure 4:**
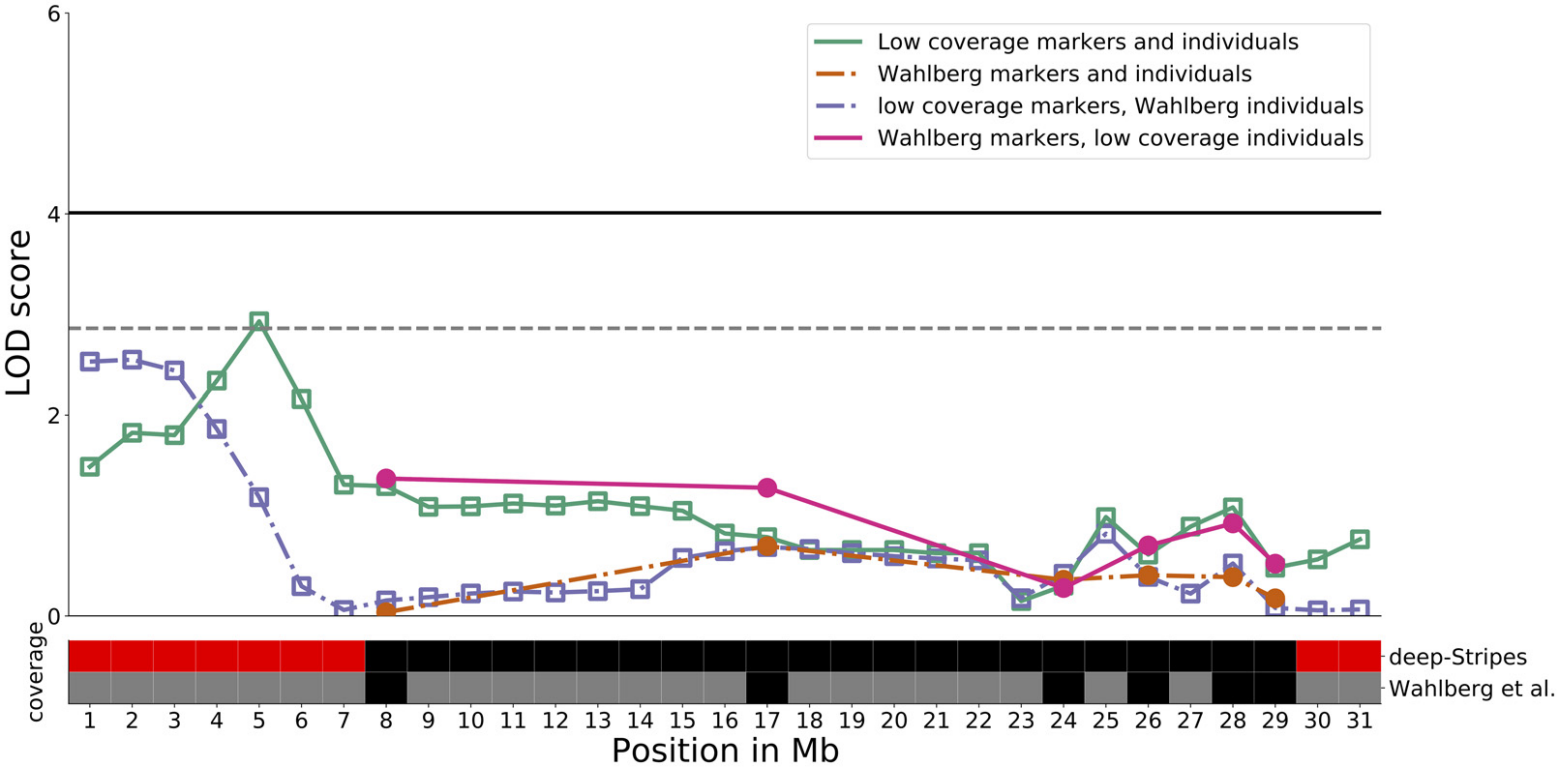
A novel QTL due to increased coverage on Chromosome 8. Upper panel: QTL scan for 56-d body weight across Chromosome 8, using the individuals from the original $F_{2}$ genome scan (Wahlberg et al., 2009, dashed lines) and all AIL individuals (solid lines) utilizing only the marker positions from Wahlberg et al. (2009); filled circles) or all low-coverage markers (empty squares). Lower panel: Chromosome 8 sectioned into 1 Mb bins, indicating whether markers are present (black, red) or not present (grey) for both low coverage data (upper row) and the marker set from the original $F_{2}$ scan by Wahlberg et al. (2009; lower row). Red highlights chromosome ends with new genotypes outside the outermost markers in Wahlberg et al. (2009).

The low coverage sequencing approach implemented here improved the genome-wide marker coverage in this population. In particular, the coverage was improved by reaching further out to the ends of almost all chromosomes, specifically 5, 6, and 8 (Figure 1). As a result, a suggestive QTL for 56-d body weight was observed on Chromosome 8 where an additional 7 Mb (0–7 Mb) were covered on its distal end (Figure 4b). The QTL peak is located on the end of the chromosome and did not extend into the part of the chromosome covered in the earlier studies (Wahlberg et al., 2009). Its effect is relatively small, and hence explains a modest amount of the variance in 56-d body weight (Table S1). Its peak location was further located approximately 20 cM outside of the most distal marker in the linkage map used by Wahlberg et al. (2009). Figure 4 illustrates how the combination of better regional coverage and gain in power from merging individuals from multiple generations in the AIL resulted in its detection.

### Doubling the Number of Genome-Wide Significant QTL Identified Triples the Amount of the Founder-Line Difference Explained for the Selected Trait

In this study, the intercross population was bred from the Virginia body weight selection lines. These two pedigreed populations were divergently selected long-term for a single trait, 56-d body weight, for 40 generations before the intercross was formed. Previous whole-genome analyses of this intercross were challenged to detect genome-wide significant QTL, finding only 1 (Jacobsson et al., 2005) or 2 (Wahlberg et al., 2009). Subsequent fine-mapping analyses focused on significant and suggestive QTL regions and selective-sweep regions (Johansson et al., 2010; Besnier et al., 2011; Pettersson et al., 2011, 2013; Sheng et al., 2015; Zan et al., 2017) Results from these studies suggested that the genetic architecture of 56-d body weight in this population was highly polygenic and that individual loci contributed small marginal effects.

The increased power in our study obtained by increasing quality and coverage of markers, as well as increasing population-size by integrating across generational data, facilitated detection of 10 additional genome-wide significant QTL associated with 56-d body weight. None had been previously identified as significant. The residual phenotypic variance explained by the mapped QTL increased from 5.3% by the 2 genome-wide significant QTL of Wahlberg et al. (2009) to 8.3% by the 12 genome-wide significant QTL detected here. The effect sizes of the QTL estimated in the population analyzed here were often lower than previous estimates, with the exception of *Growth1*. The reduced effect size for *Growth9* was not surprising, given that Chromosome 7, and *Growth9* in particular, were involved in epistatic interactions (Pettersson et al., 2011) and is likely a complex region. For *Growth6*, while the estimate is lower than that in Wahlberg et al. (2009), the sum of all significant and suggestive QTL on Chromosome 4 was larger (91.7 g compared to 79.1 g) than the estimate for the 2 earlier mapped QTL on Chromosome 4 (*Growth6* and *Growth7;* Jacobsson et al., 2005). Our study thus confirms previous suggestive evidence obtained in studies of related growth phenotypes.

Additionally, our study enabled fine-mapping them into multiple independent regions previously associated with 56-d body weight found by selection-scans (Zan et al., 2017). In total, out of 11 markers significant in Zan et al. (2017), all but 2 were situated in QTLs passing the 10% FDR threshold here. Further, the considerable overlap between the QTL from this study and the previously identified sweep regions from Johansson et al. (2010) , supports the hypothesis that the sweeps result from selection acting on QTL rather than being the result of drift.

Overall, the effects of the significant QTL contributed 37.4% of the founder-line difference (Table S2), which is >3 times the founder-line difference explained by the significant QTL in Wahlberg et al. (2009). The QTL significant at a 10% FDR-threshold explained more than 84% of the difference between the founder lines. A caveat with this estimation is the large fraction of the genome covered by these markers, and hence the approach used here to estimate the effect size of individual loci likely contributed to a slight overestimation from residual linkage between QTL and tagging of linked loci. The estimates might also be affected by a “winners curse,” as the effect sizes were estimated using the same individuals as in the QTL mapping. We consider a likely range of the estimate to be between this estimate and that of the joint fit (59%). This due to the joint fit likely being an underestimate, for the same reasons as described above.

Further, 30.3% of the bins on the covered autosomal chromosomes were statistically associated with the phenotype after lenient adjustment for multiple testing. This means that any overlap between the extended QTL and the selective sweep regions should be interpreted with caution. Still, this supports the interpretation of a high polygenicity of the investigated trait, and it is reasonable to believe that these regions were undergoing true and detectable selection, given the strong single trait artificial selection regime in the selected lines (Siegel, 1962; Dunnington and Siegel, 1996; Márquez et al., 2010; Dunnington et al., 2013).

### Added Value by Increased Genome-Wide Marker Coverage

Genotyping by sequencing has provided opportunities to both increase marker density and decrease the cost for genotyping. Strategies based on low-coverage sequencing approaches open cost-efficient opportunities to re-analyze existing experimental populations. Here, we demonstrated one such approach targeted to deep outbred intercrosses. This approach increased the overall marker coverage of the chicken genome from 93% (Wahlberg et al., 2009), as estimated as coverage of the autosomal genetic map, to 1,058 Mb (99.3% of 1,065 Mb; Genome Reference Consortium, 2018) with a mean density of 102 line-informative markers per Mb.

Beyond Markers that are fixed and therefore always informative for line-origin, outbred founder lines have many more markers that can be line-informative since they are fixed between the HWS and LWS founders contributing to the ancestry of an individual, despite still segregating within the lines. However, as the number of these markers decreases with the increase in unique ancestors contributing to each individual in later generations, the depth of generations one can investigate with this can be limited by the number of founding individuals as well as the size of each generation. Thus, it is important that the founder lines are sufficiently divergent to provide a minimum resolution via fixed markers. However, because the nonphysical window size of the imputation process does not sacrifice higher resolution in divergent regions, it is likely that any cross between lines with a significant genetic component to their phenotypic divergence will have sufficient coverage with informative markers in regions of interest. For the AIL used in this study, it is likely that the resolution is not limited by the marker coverage, but rather by both the 1 Mb binning approach and the 50/200 marker sliding window imputation of founder genotypes. This is because there were enough accumulated recombinations in the later generations that were lost through these approaches. While these were chosen for robustness and provided an appropriate resolution/power trade-off across all individuals, modifying these parameters could provide added opportunities for fine mapping.

The increased marker density also increased the information content throughout the genome, which further increased power in the QTL analyses. In particular, coverage was extended at the ends of the larger chromosomes. This led to locating a novel suggestive QTL on Chromosome 8 that was primarily due to increase in coverage, though the increase in power helped elevate and define the peak of the QTL. It was seen in the full marker set using only the *F_2_* population, but without a definitive peak. This is likely due to a lack of recombination events distal to the peak in the *F_2_* population, though the LOD scores for the outermost 3 Mb were above the 5% permutation threshold for chromosome-wide significance.

In addition, two additional small linkage groups were also covered. The minor regions of the genome that remain are present in scaffolds containing too few markers for reliable genotype estimation using the *Stripes* pipeline. Additional work beyond this study is needed to estimate the genotype in these regions using alternative genotyping or bioinformatics approaches before they can be included in QTL mapping studies.

### Integrating AIL Data

Integrating data across an AIL population provides value to the QTL scan by helping to map new QTL and refining the resolution and explanatory power of existing ones. This is particularly so when intermediate generations already exist due to previous attempts in fine mapping. At less than 1 EUR/sample for library preparation and 10 EUR/sample for library preparation and sequencing (Zan et al., 2019), our approach is cost-effective in enhancing the statistical power to dissect complex traits from potentially any experimental population or selection experiment.

For AIL populations with smaller intermediate generations and multiple siblings or half sibs, correcting generation with a mixed or fixed effect model will likely result in overcorrection due to the correlation between generations and kinship. Standardization using z-scores provides an acceptable trade-off between overcorrecting and confidence in accounting for generational batch effects.

In conclusion, this study represents a more comprehensive study of the individual loci forming the genetic basis of the highly polygenic, long-term selection responses on 56-d body weight in the Virginia chicken lines, to date. It contributes not only to our current understanding of the genetic basis of body weight in chickens, but also provides a solid methodological foundation to further investigate the genetic architecture of complex traits in populations with similar designs.

## Appendix Supplementary materials

Image, application 1

Image, image 2

Image, application 3

Image, application 4

Image, application 5

Image, application 6
