# Supplementary material for: Low-coverage sequencing in a deep intercross of the Virginia body weight lines provides insight to the polygenic genetic architecture of growth: novel loci revealed by increased power and improved genome-coverage
Source: Poult Sci. 2022 Oct 1;102(5):102203. doi: 10.1016/j.psj.2022.102203 (PMC10024170; doi:10.1016/j.psj.2022.102203)
Supplement: Supplementary file 1 [file mmc1.docx]

This submission has an associated preprint on biorxiv.org:

<https://www.biorxiv.org/content/10.1101/2021.07.19.451141v1>

**doi:** https://doi.org/10.1101/2021.07.19.451141
