# Supplementary figures and images for: Low-coverage sequencing in a deep intercross of the Virginia body weight lines provides insight to the polygenic genetic architecture of growth: novel loci revealed by increased power and improved genome-coverage
Source: Poult Sci. 2022 Oct 1;102(5):102203. doi: 10.1016/j.psj.2022.102203 (PMC10024170; doi:10.1016/j.psj.2022.102203)

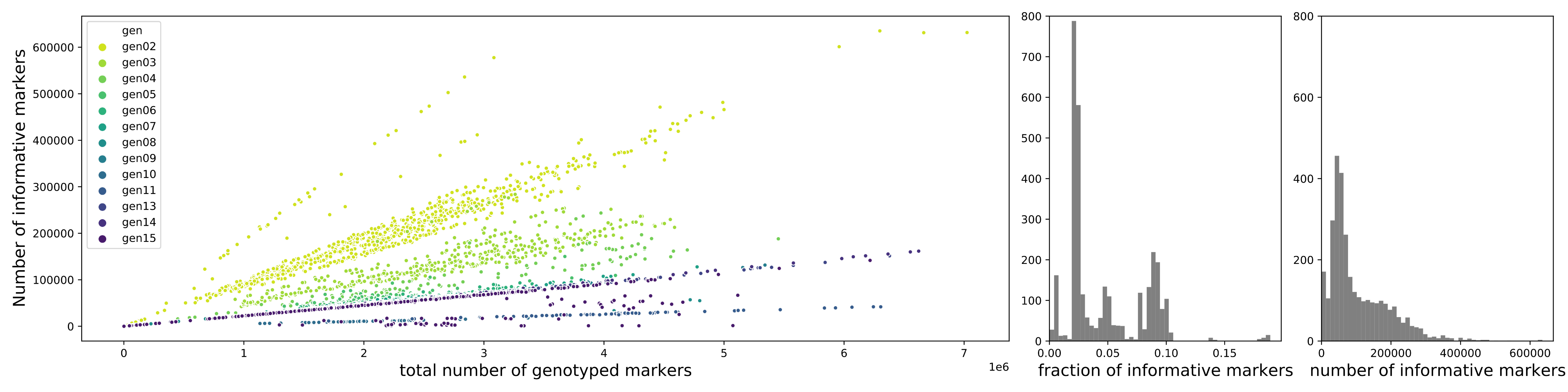

Supplement: Supplementary file 2 [file mmc2.jpg]
