# Supplementary material for: Low-coverage sequencing in a deep intercross of the Virginia body weight lines provides insight to the polygenic genetic architecture of growth: novel loci revealed by increased power and improved genome-coverage
Source: Poult Sci. 2022 Oct 1;102(5):102203. doi: 10.1016/j.psj.2022.102203 (PMC10024170; doi:10.1016/j.psj.2022.102203)
Supplement: Supplementary file 3 [file mmc3.docx]

**Table S1.** All suggestive and significant QTL peaks for 56-day body weight and overlaps with earlier reported QTL in this population

|  | Chromosome | Position(Mb) | a(SE) | %var | lod | QTL | previous |
| --- | --- | --- | --- | --- | --- | --- | --- |
|  |  |  |  |  |  |  |  |
|  | 1 | 56 | 19.6(4.0) | 0.43 | **5.79** |  |  |
|  | 1 | 171 | 31.6(4.1) | 1.21 | **13.08** | Growth1 | Zan 2017,  Wahlberg 2009,  Jacobsson 2005 |
|  | 2 | 99 | 13.5(4.0) | 0.25 | 2.96 | Growth3 | Jacobsson 2005 |
|  | 2 | 113 | 16.6(4.0) | 0.14 | 3.65 | Growth3 | Zan 2017,  Jacobsson 2005 |
|  | 3 | 35 | 11.6(4.3) | 0.27 | 2.87 | Growth4 | Zan 2017,  Jacobsson 2005 |
|  | 3 | 74 | 20.3(4.2) | 0.31 | **5.41** | Growth4 | Jacobsson 2005 |
|  | 4 | 11 | 20.7(4.4) | 0.28 | **5.29** | Growth6 | Zan 2017,  Wahlberg 2009 |
|  | 4 | 23 | 20.4(4.0) | 0.07 | **6** | Growth7 ? | Jacobsson 2005 |
|  | 4 | 36 | 25.6(4.2) | 0.45 | **8.2** | Growth7 ? | Jacobsson 2005 |
|  | 4 | 70 | 14.6(3.9) | 0.2 | 3.83 | Growth7 ? | Zan 2017,  Jacobsson 2005 |
|  | Z | 1 | 12.9(3.5) | 0.11 | 2.95 |  |  |
|  | Z | 10 | 12.8(3.8) | 0.03 | 3.22 |  |  |
|  | Z | 34 | 14.0(3.4) | 0.05 | 3.8 |  |  |
|  | 5 | 30 | 20.9(4.3) | 0.31 | **5.22** | Growth8 | Jacobsson 2005 |
|  | 7 | 4 | 5.2(4.3) | 0.28 | 3.04 |  |  |
|  | 7 | 21 | 25.6(4.0) | 1.26 | **9.6** | Growth9 | Zan 2017,  Wahlberg 2009,  Jacobsson 2005 |
|  | 8 | 4 | -7.1(4.2) | 0.3 | 2.93 |  |  |
|  | 10 | 9 | 22.3(3.9) | 0.27 | **7.25** |  | Zan 2017 |
|  | 10 | 21 | 15.8(4.3) | 0.1 | 3.86 |  |  |
|  | 11 | 7 | 13.2(4.0) | 0.32 | **4.22** |  |  |
|  | 13 | 12 | 19.6(3.9) | 0.32 | **5.83** | Growth10 | Jacobsson 2005 |
|  | 14 | 6 | 14.0(4.1) | 0.16 | 3.41 |  |  |
|  | 14 | 16 | 16.0(4.3) | 0.01 | 3.15 |  |  |
|  | 20 | 11 | 16.3(4.3) | 0.5 | 3.23 | Growth12 | Zan 2017,  Wahlberg 2009 |
|  | 23 | 6 | 10.9(4.3) | 0.47 | **4.32** |  | Zan 2017 |
|  |  |  |  |  |  |  |  |
|  |  |  | a=407 |  |  |  |  |
|  |  |  | 2a=814 |  |  |  |  |
