# Supplementary material for: Low-coverage sequencing in a deep intercross of the Virginia body weight lines provides insight to the polygenic genetic architecture of growth: novel loci revealed by increased power and improved genome-coverage
Source: Poult Sci. 2022 Oct 1;102(5):102203. doi: 10.1016/j.psj.2022.102203 (PMC10024170; doi:10.1016/j.psj.2022.102203)
Supplement: Supplementary file 4 [file mmc4.docx]

**Table S2:** Mean bodyweight and number of individuals per generation

| generation | individuals | BW8 - mean(stdev)g |
| --- | --- | --- |
| HWS-$F_{0}$ | 29 | 1522(36) |
| LWS-$F_{0}$ | 30 | 181(5) |
| HWS-LWS difference |  | 1341 |
| $F_{2}$ | 930 | 626(186) |
| $F_{3}$ | 429 | 696(170) |
| $F_{4}$ | 112 | 593(131) |
| $F_{5}$ | 118 | 656(155) |
| $F_{6}$ | 88 | 770(168) |
| $F_{7}$ | 41 | 661(178) |
| $F_{8}$ | 292 | 375(122) |
| $F_{9}$ | 50 | 710(186) |
| $F_{10}$ | 60 | 674(175) |
| $F_{11}$ | 87 | 579(165) |
| $F_{12}$ | 76 | 603(180) |
| $F_{14}$ | 32 | 730(192) |
| $F_{15}$ | 818 | 613(162) |
| $F_{16}$ | 89 | 650(174) |
| $F_{17}$ | 45 | 576(165) |
| $F_{18}$ | 87 | 528(148) |
| all | 3354 | 614(188) |
