# Supplementary material for: Low-coverage sequencing in a deep intercross of the Virginia body weight lines provides insight to the polygenic genetic architecture of growth: novel loci revealed by increased power and improved genome-coverage
Source: Poult Sci. 2022 Oct 1;102(5):102203. doi: 10.1016/j.psj.2022.102203 (PMC10024170; doi:10.1016/j.psj.2022.102203)
Supplement: Supplementary file 5 [file mmc5.docx]

**Table S3:** All markers reaching significance after false-discovery-rate adjustment with alpha=10%

|  |  |  |  |  |  |
| --- | --- | --- | --- | --- | --- |
|  | Chromosome | Position(Mb) | a(SE) in gram | % variance explained | LOD |
|  | 1 | 35 | 10.32(4.156) | 0.11947 | 1.765 |
|  | 1 | 56 | 19.62(4.038) | 0.4807 | 5.788 |
|  | 1 | 79 | 2.36(4.191) | 0.20871 | 1.799 |
|  | 1 | 93 | -12.34(4.009) | 0.19455 | 2.144 |
|  | 1 | 132 | 11.27(3.875) | 0.1573 | 2.457 |
|  | 1 | 171 | 31.59(4.059) | 1.11277 | 13.077 |
|  | 1 | 185 | -3.88(4.077) | 0.08036 | 1.731 |
|  | 2 | 5 | 10.55(3.966) | 0.117 | 1.732 |
|  | 2 | 22 | -10.7(4.064) | 0.17337 | 1.695 |
|  | 2 | 37 | 4.3(4.016) | 0.26358 | 2.06 |
|  | 2 | 58 | 10.95(4.229) | 0.09418 | 2.513 |
|  | 2 | 62 | 11.87(4.118) | 0.01154 | 2.707 |
|  | 2 | 99 | 13.47(3.97) | 0.1638 | 2.963 |
|  | 2 | 113 | 16.57(4.039) | 0.12504 | 3.651 |
|  | 2 | 141 | 14.19(3.948) | 0.12695 | 2.808 |
|  | 3 | 35 | 11.63(4.268) | 0.2559 | 2.867 |
|  | 3 | 74 | 20.35(4.165) | 0.2721 | 5.408 |
|  | 4 | 11 | 20.71(4.367) | 0.16289 | 5.294 |
|  | 4 | 23 | 20.44(3.989) | 0.05897 | 6.003 |
|  | 4 | 37 | 24.82(4.213) | 0.63225 | 7.631 |
|  | 4 | 71 | 14.39(3.932) | 0.25961 | 3.623 |
|  | 4 | 91 | 11.35(4.241) | 0.2026 | 2.468 |
|  | 5 | 3 | 5.73(4.055) | 0.29812 | 2.366 |
|  | 5 | 11 | 12.24(3.962) | 0.15097 | 2.1 |
|  | 5 | 17 | 9.34(4.222) | 0.09418 | 1.82 |
|  | 5 | 30 | 20.89(4.27) | 0.22515 | 5.225 |
|  | 6 | 10 | 10.95(4.029) | 0.08556 | 1.629 |
|  | 7 | 4 | 5.22(4.256) | 0.30981 | 3.042 |
|  | 7 | 21 | 25.56(3.98) | 0.97001 | 9.599 |
|  | 8 | 4 | -7.1(4.232) | 0.25772 | 2.93 |
|  | 10 | 9 | 22.3(3.912) | 0.29332 | 7.249 |
|  | 11 | 7 | 13.17(4.036) | 0.2799 | 4.223 |
|  | 13 | 12 | 19.62(3.92) | 0.25145 | 5.826 |
|  | 14 | 6 | 14.04(4.116) | 0.14015 | 3.408 |
|  | 14 | 16 | 16.0(4.274) | 0.02953 | 3.149 |
|  | 18 | 3 | -9.74(4.036) | 0.22344 | 1.689 |
|  | 19 | 9 | 8.04(4.08) | 0.12524 | 1.858 |
|  | 20 | 11 | 16.34(4.27) | 0.60823 | 3.231 |
|  | 21 | 6 | 9.59(3.496) | 0.26196 | 2.075 |
|  | 23 | 6 | 10.86(4.284) | 0.35913 | 4.318 |
|  | 28 | 0 | 7.9(4.252) | 0.20064 | 2.47 |
|  | 33 | 4 | -12.35(4.411) | 0.25283 | 2.138 |
|  |  |  |  |  |  |
|  |  |  | a = 565.44 |  |  |
|  |  |  | 2a = 1130.87 |  |  |
|  |  |  |  |  |  |
