# Supplementary material for: Low-coverage sequencing in a deep intercross of the Virginia body weight lines provides insight to the polygenic genetic architecture of growth: novel loci revealed by increased power and improved genome-coverage
Source: Poult Sci. 2022 Oct 1;102(5):102203. doi: 10.1016/j.psj.2022.102203 (PMC10024170; doi:10.1016/j.psj.2022.102203)
Supplement: Supplementary file 6 [file mmc6.docx]

**Table S4:** joint-fit estimates for all QTLs passing 10% FDR

|  |  |  |  |  |  |
| --- | --- | --- | --- | --- | --- |
|  | Chromosome | Position(Mb) | a(SE) in gram |  |  |
|  | 1 | 35 | 8.14(4.057) |  |  |
|  | 1 | 56 | 15.16(4.0) |  |  |
|  | 1 | 79 | 11.89(4.701) |  |  |
|  | 1 | 93 | -12.5(5.197) |  |  |
|  | 1 | 132 | -9.45(4.198) |  |  |
|  | 1 | 171 | 4.76(3.928) |  |  |
|  | 1 | 185 | 25.68(4.016) |  |  |
|  | 2 | 5 | -1.67(4.106) |  |  |
|  | 2 | 22 | 8.11(3.935) |  |  |
|  | 2 | 37 | -8.33(4.112) |  |  |
|  | 2 | 58 | 6.52(3.991) |  |  |
|  | 2 | 62 | 5.58(4.591) |  |  |
|  | 2 | 99 | 2.46(4.417) |  |  |
|  | 2 | 113 | 8.7(3.999) |  |  |
|  | 2 | 141 | 8.86(4.122) |  |  |
|  | 3 | 35 | 8.54(3.928) |  |  |
|  | 3 | 74 | 8.17(4.107) |  |  |
|  | 4 | 11 | 12.12(4.064) |  |  |
|  | 4 | 23 | 10.63(4.428) |  |  |
|  | 4 | 37 | 4.24(4.248) |  |  |
|  | 4 | 71 | 20.65(4.266) |  |  |
|  | 4 | 91 | 11.09(3.843) |  |  |
|  | 5 | 3 | 6.77(4.191) |  |  |
|  | 5 | 11 | -9.39(4.31) |  |  |
|  | 5 | 17 | 8.88(4.333) |  |  |
|  | 5 | 30 | 0.45(4.606) |  |  |
|  | 6 | 10 | 13.04(4.506) |  |  |
|  | 7 | 4 | 6.63(3.988) |  |  |
|  | 7 | 21 | -7.39(4.362) |  |  |
|  | 8 | 4 | 23.37(3.98) |  |  |
|  | 10 | 9 | -5.31(4.149) |  |  |
|  | 11 | 7 | 11.66(3.899) |  |  |
|  | 13 | 12 | 8.46(3.916) |  |  |
|  | 14 | 6 | 11.2(3.861) |  |  |
|  | 14 | 16 | 7.83(4.795) |  |  |
|  | 18 | 3 | 3.39(4.989) |  |  |
|  | 19 | 9 | -9.35(3.907) |  |  |
|  | 20 | 11 | 3.21(4.042) |  |  |
|  | 21 | 6 | 19.98(4.2) |  |  |
|  | 23 | 6 | 10.51(3.378) |  |  |
|  | 28 | 0 | 2.06(4.247) |  |  |
|  | 33 | 4 | 1.43(4.183) |  |  |
|  |  |  |  |  |  |
|  |  |  | a = 396.45 |  |  |
|  |  |  | 2a = 792.89 |  |  |
|  |  |  |  |  |  |
